# Audit on use of PRN (pro re nata) psychotropic medication for behavioural disturbance in individuals with intellectual disability in the community

**DOI:** 10.1192/bjo.2021.240

**Published:** 2021-06-18

**Authors:** Asha Dhandapani, Sathyan Soundararajan, Claire Jones

**Affiliations:** BCUHB

## Abstract

**Aims:**

Psychotropic medication is commonly used in people with Intellectual disabilities (ID). This may be attributed in part to an increased prevalence of mental illness in this population and the presence of challenging behaviour which has been shown to increase rates of prescribing. Whilst there are a number of studies looking at regularly prescribed medication there are few studies on “as and when” required (PRN) medication.

Psychotropic medication continues to be used to manage behavioural disturbances in people with ID. Where there is no clear cut psychiatric illness, the role of psychotropic medication is an adjunct to a comprehensive multimodal treatment plan.

The aim is to find out if prn psychotropic medication for behavioural disturbance is being used appropriately and safely in these individuals.

**Method:**

Files and PRN protocols of individuals known to be using prn psychotropic medications for the management of acute episodes of agitation and behavioural problems and supported by professional staff teams was studied.

We collected the data by contacting the residential homes, carers, Collecting details from case notes, from the Staff nurse who made the protocol for their patients

A questionnaire based on the standards mentioned above was developed and files and prn protocols were marked against these standards.

**Result:**

The standards from the medical file were 100 % achieved. Thus indicating the importance of the psychotropic prn medication and documentation of the same.

However, the protocol that needs to be with the patient/carers had some lacuna/deficits. Overall only in 53% of the case, standards were achieved. This needs to be highlighted to the team.

The Audit gave an insight into what needs to be improved.

THE FOLLOWING AREAS NEEDED IMPROVEMENT

1. There should be a prn protocol/ similar instruction to the staff about the use of prn medication(written by appropriately trained professional)
2. Prn protocol should be accessible to direct care staff
3. There should be a description of when to use the prn medication
4. There should be a description of what non-pharmacological de-escalation methods ought to be tried before using prn/ is there a detailed behaviour support plan available
5. Protocol should describe what the medication is expected to do
6. Protocol should describe the minimum time between doses if the first dose has not worked
7. Protocol should state the maximum dose in 24 hour period
8. Use of prn should be recorded

**Conclusion:**

I hope this audit will help in improving the patient care with the right psychotropic prn medication, with correct doses and further details as mentioned in the standards of the protocol.

We also hope to ensure that in our area, prn psychotropic medication used for agitation and behavioural disturbance is used safely, appropriately and consistently by staff teams. This would be in accordance with the guidelines.

